# Putative neural and endocrine control of thermal acclimation in fish

**DOI:** 10.1093/conphys/coaf042

**Published:** 2025-06-17

**Authors:** Robine H J Leeuwis, Rachael Morgan, Anna H Andreassen, Lorena Silva-Garay, Zara-Louise Cowan, Eirik R Åsheim, Jeremy De Bonville, Sandra A Binning, Graham D Raby, Fredrik Jutfelt

**Affiliations:** Department of Biology, Norwegian University of Science and Technology, Høgskoleringen 5, Trondheim, 7034, Norway; Department of Biological Sciences, University of Bergen, Thormøhlens gate 53 A/B, Bergen, 5006, Norway; Department of Biology, Norwegian University of Science and Technology, Høgskoleringen 5, Trondheim, 7034, Norway; DTU Aqua, National Institute of Aquatic Resources, Technical University of Denmark, Henrik Dams Allé Building 202, Kongens Lyngby, 2800, Denmark; Department of Biology, Norwegian University of Science and Technology, Høgskoleringen 5, Trondheim, 7034, Norway; Department of Biology, Norwegian University of Science and Technology, Høgskoleringen 5, Trondheim, 7034, Norway; Natural Resources Institute Finland (Luke), Paavo Havaksen tie 3, Oulu, 90570, Finland; Department of Aquatic Resources, Swedish University of Agricultural Sciences, Stångholmsvägen 2, Drottningholm, 178 93, Sweden; Department of Biological Sciences, University of Montréal, 1375 Av. Théresè-Lavoie-Roux, Montréal, QC H2V 0B3, Canada; Department of Biological Sciences, University of Montréal, 1375 Av. Théresè-Lavoie-Roux, Montréal, QC H2V 0B3, Canada; Department of Biology, Trent University, 1600 West Bank Drive, Peterborough, ON K9L 0G2, Canada; Department of Biology, Norwegian University of Science and Technology, Høgskoleringen 5, Trondheim, 7034, Norway; Department of Biology and Environmental Sciences, Faculty of Natural Sciences, University of Gothenburg, Kristineberg Center, Kristineberg 566, Fiskebäckskil, 451 78, Sweden

**Keywords:** Ectotherm, metabolism, respirometry, thermal plasticity, thermal tolerance, thermoreception

## Abstract

Fishes can acclimate to a range of temperatures. However, the signalling factors controlling thermal acclimation are not well understood. Here, in two experiments, we examined the putative roles of plasma-borne factors (e.g. hormones) and skin thermoreception in the acclimation process. In experiment 1, 16°C-acclimated Atlantic cod (*Gadus morhua*) were subjected to a transfusion treatment by injecting plasma from 8°C (cold), 16°C (control) or 21°C (warm) acclimated cod, 10 times over four days. Plasma was collected from donor cod that were 24 h into their acclimation. In experiment 2, 16°C-acclimated goldsinny wrasse (*Ctenolabrus rupestris*) were exposed to an immersion treatment consisting of 10 s immersions in an 8°C (cold), 16°C (control) or 24°C (warm) water bath, repeated five times daily for five days. These brief immersions allowed for changes to skin temperature but not deeper tissues. Following these treatments, we measured the critical thermal maximum (CT_max_) of all fish and the standard metabolic rate (SMR) in cod. Neither the immersions nor transfusions affected fish CT_max_. However, the SMR was elevated in cod receiving plasma from cold-acclimated donors, suggesting that circulating molecules transferred from donors had initiated metabolic compensation in recipients. Thyroid hormone plasma levels were not different amongst acclimated donors and thus appear not to have been involved in the metabolic compensation. Our experiments found no evidence that brief, repeated cutaneous exposures to temperature changes can trigger acclimation, but do demonstrate a potential role of haematological endocrine control in metabolic acclimation, although further experiments will be required to investigate this process.

## Introduction

All ectothermic animals need to cope with changes in body temperature when exposed to thermal variation in their environment (Somero *et al.,* 2016). Because temperature directly affects molecular movements, it can alter the rate of biochemical reactions, protein structure and function and membrane fluidity, which together impact organ function and whole-organism performance (Schulte, 2015; Jutfelt *et al.,* 2024; Schulte, 2024). An important strategy that enables ectotherms to deal with body temperature changes is thermal acclimation (or thermal compensation), a process involving adjustments to biochemistry, physiology and morphology that counteract or buffer the direct physicochemical effects of temperature (Seebacher *et al.,* 2015). Thermal acclimation allows animals to optimize biological functions at the new temperature and ultimately to maintain performance across a wider thermal range (Angilletta, 2009). For instance, acute cooling slows down an organism’s metabolism, but cold acclimation can partially overcome this effect by increasing mitochondrial respiration capacity via higher mitochondrial volume density, enzyme activity and electron transport system rates, which in turn increases metabolic rate (Egginton and Sidell, 1989; Dhillon and Schulte, 2011; Wolfe *et al.,* 2020; Cominassi *et al.,* 2022; Reeve *et al.,* 2022). Conversely, acute warming can threaten protein integrity, whereby warm acclimation can lead to elevated tissue levels of thermoprotective heat shock proteins (HSPs), which help to sustain cellular protein function (Dietz and Somero, 1992; Morgan *et al.,* 2022).

Many physiological adjustments occurring during thermal acclimation (also known as thermal plasticity) have been documented in ectotherms (Huey and Bennett, 1990; Glanville and Seebacher, 2006; Seebacher *et al.,* 2015; Somero *et al.,* 2016; Leeuwis and Gamperl, 2022; Jutfelt *et al.,* 2024). However, the neurosensory and endocrine initiation and control of the thermal acclimation process are poorly understood, and few studies have explored this topic, especially in fishes. In ectothermic vertebrates, the brain and central nervous system may be responsible for the induction and regulation of thermal acclimation including the direction and magnitude of the response (Seebacher, 2009). Alternatively, thermal acclimation could occur in a peripheral, diffuse manner at the cellular and tissue level, whereby each cell directly responds to the local temperature without centralized neural or hormonal signalling (Klicka, 1965; Horowitz, 2001; Logan and Somero, 2011).

In all vertebrate phylogenetic groups, the brain receives information about the core body (internal) temperature through receptors in the brain itself (preoptic area/anterior of the hypothalamus), and about the environmental (external) temperature via peripheral thermosensitive neurons in the trigeminal and dorsal root ganglion that innervate the skin and visceral organs (Patapoutian *et al.,* 2003; Gracheva and Bagriantsev, 2015; Wang and Siemens, 2015; Haesemeyer, 2020; Kashio, 2021). In fishes, the pineal gland and the retina also appear to be part of the thermoperceptive circuitry (Nisembaum *et al.,* 2022). Integrated information relayed by these thermosensory neural pathways may inform the brain on the need for thermal acclimation in ectotherms.

Thermosensation is facilitated by temperature-activated transient receptor potential (thermoTRP) ion channels, which are conserved across the animal kingdom (Somero *et al.,* 2016). Each thermoTRP channel is activated by a specific temperature threshold, from cold temperatures (cold sensors, e.g. TRPM8), intermediate temperatures (e.g. TRPV3), to warm temperatures (warm sensor, e.g. TRPV1) (Patapoutian *et al.,* 2003; Gracheva and Bagriantsev, 2015; Wang and Siemens, 2015; Hoffstaetter *et al.,* 2018; García-Ávila and Islas, 2019; Kashio, 2021). The sensitivity of thermoTRPs differs amongst species and is generally tuned to respond to temperatures outside the species’ preferred/optimal range (Gracheva and Bagriantsev, 2015; Somero *et al.,* 2016; Hoffstaetter *et al.,* 2018). In mammals, TRPV1 triggers a (localized) cellular heat shock response by directly increasing HSP expression (Bromberg *et al.,* 2013). In fishes, a link between TRPV1 thermoperception and the HSP response may also exist (Jerônimo *et al.,* 2017), although evidence is still lacking.

If central control of thermal acclimation is the prominent mechanism, then hormones or other molecules circulating in plasma would likely play a role in signalling between the brain and peripheral tissues. Indeed, thyroid hormone (TH) is a putative regulator of thermal plasticity in ectotherms (Hoar and Eales, 1963; Prosser *et al.,* 1991; Little *et al.,* 2013; Little and Seebacher, 2014; Zak *et al.,* 2017; Zak and Manzon, 2019; Le Roy and Seebacher, 2020; Little, 2021). TH also regulates a variety of other processes, such as growth, development, metamorphosis, metabolism, osmoregulation and reproduction in fishes (Besson *et al.,* 2020; Deal and Volkoff, 2020), and metabolic rate and heat production in mammals (Silva, 1995). While thyroxine (T_4_) is the predominant circulating form of TH, 3,5,3′-triiodothyronine (T_3_) is considered more biologically active (Cyr *et al.,* 1998; Little *et al.,* 2013; Deal and Volkoff, 2020).

The nature of the regulatory control of thermal acclimation by TH in ectotherms has not been elucidated. For example, it is unclear if TH primarily mediates cold or warm acclimation, or both. In cold-acclimated zebrafish (*Danio rerio*), pharmacologically induced hypothyroidism impaired performance traits (metabolic rates, swimming performance, maximum heart rate), which were restored by 3,5-diiodothyronine (T_2_) and T_3_ supplementation, while hypothyroidism did not affect performance in warm-acclimated zebrafish (Little *et al.,* 2013; Little and Seebacher, 2014). In contrast, in cold-acclimated lake whitefish (*Coregonus clupeaformis*), induction of hyperthyroidism via exogenous T_4_ exposure had little effect on metabolic enzyme expression and activity, whereas in warm-acclimated whitefish, hyperthyroidism strongly stimulated metabolic enzyme expression and activity in the liver and heart (Zak *et al.,* 2017; Zak and Manzon, 2019). Mechanistically, TH may act centrally via the preoptic area of the hypothalamus, which could result in increases in heart rate, or act directly on peripheral tissues, resulting in metabolic remodelling in tissues such as cardiac and skeletal muscle and the liver (Prosser *et al.,* 1991; Little, 2021). These effects may help to explain the whole-organism changes that are typically evident following thermal acclimation in ectotherms.

For this study, we designed two experiments to examine both the neurosensory and endocrine control of thermal acclimation in fishes. In experiment 1, we investigated the role of circulating regulatory factors (e.g. hormones) in thermal acclimation, using Atlantic cod (*Gadus morhua*) as the experimental model. Since the hormones involved in thermal acclimation are still relatively unknown, we transfused plasma from cold-acclimated or warm-acclimated donors to control-acclimated recipients to confirm the presence of an endocrine signal for thermal acclimation and whether it can be transmitted—similar to the work by Enok *et al.* (2012) in pythons. Furthermore, we measured T_3_ levels in donor plasma as the main candidate hormonal regulator. We assessed the thermal acclimation response in donors and recipients by measuring critical thermal tolerance (CT_max_), which is a commonly used metric to quantify thermal plasticity in ectotherms (Ruthsatz *et al.,* 2024). We also measured the standard metabolic rate (SMR) in recipients because compensatory adjustments in this metabolic trait have often been reported in cold or warm acclimating fishes (Gräns *et al.,* 2014; Sandblom *et al.,* 2014; Cominassi *et al.,* 2022). We hypothesized that hormones and other plasma-borne mediators are involved in orchestrating thermal acclimation. Therefore, we predicted that cod infused with plasma from cold-acclimated donors would display a reduced CT_max_ and increased SMR. Conversely, we predicted that transfusions of plasma from warm-acclimated donors would increase CT_max_ and reduce SMR.

In experiment 2, we investigated the role of neural sensing of the external temperature in goldsinny wrasse (*Ctenolabrus rupestris*). An early experiment suggested that repeated intermittent heating can cause warm acclimation in fish (Loeb and Wasteneys, 1912). We used short, repeated immersions in cold or warm water to expose the skin and other superficial tissues—but not the core of the body—to cooling or warming, in order to test whether this induces a thermal acclimation response. Thus, the environmental temperature change would be primarily perceived by cutaneous thermoreceptors, and not directly by thermoreceptors in the brain. As in experiment 1, we assessed the thermal acclimation response with CT_max_. We hypothesized that repeated cutaneous exposure to warm or cold temperatures would initiate thermal acclimation in wrasse, whereby warm-exposed fish would have a higher CT_max_ and vice-versa for cold-exposed fish.

Atlantic cod and goldsinny wrasse are suitable species for these experiments, given both are widespread in shallow-water habitats where they encounter substantial thermal variability (Sayer and Davenport, 1996; Righton *et al.,* 2010; Perry *et al.,* 2024). Furthermore, cod and goldsinny play important ecological roles in coastal marine environments and have significant socio-economic value due to their relevance to aquaculture, and commercial and recreational fisheries (Sayer and Davenport, 1996; Hutchings and Rangeley, 2011; Kjesbu *et al.,* 2014; Nardi *et al.,* 2021; Perry *et al.,* 2024). The intention of this study is to provide fundamental knowledge on the thermal biology of these two Atlantic species, which could support sustainable management and conservation efforts as well as advance our understanding of thermal acclimation in ectotherms in general.

## Materials and Methods

### Ethics statement

Experiments were conducted at the Kristineberg Centre for Marine Research and Innovation, University of Gothenburg, located on the west coast of Sweden. Current national legislation on animal welfare was followed, and sampling procedures and experimental manipulations were reviewed and approved by the Swedish Board of Agriculture (licence nr. 5.8.18–8955/2022 [permit held by Fredrik Jutfelt]).

### Animal collection and husbandry

Atlantic cod (of unknown sex, age, and life stage) were cage-caught by local fishers in the waters off Lysekil (58.27556 N, 11.43553 E), and transported in seawater-filled buckets by car (on 12 and 15 June 2022) to the research station (transportation time ~ 15 min). Large cod (>15 cm) were distributed into two ~ 1440 L tanks (150 × 120 × 80 cm [L × W × H]), and small cod (<15 cm) into two circular ~ 127 L tanks (inner diameter × height of 60 × 45 cm). Cod were fed *Pandalus borealis* shrimp once daily to satiation.

**Table 1:** Body morphometrics and sample sizes of Atlantic cod (*Gadus morhua*) and goldsinny wrasse (*Ctenolabrus rupestris*) within the different treatment groups in the study

**Species**	**Experiment**	**Treatment**	**Measurements**	**Group**	***n***	**Mass (g)**	**Standard length (cm)**
Atlantic cod	1	Transfusion (recipients)	SMR, CT_max_	Cold	5	164.8 ± 37.0	23.6 ± 2.8
				Control	6	178.7 ± 47.9	23.5 ± 2.3
				Warm	6	184.0 ± 65.8	23.9 ± 3.0
Atlantic cod	1	Acclimation (donors)	Plasma T_3_	Cold	9	405.9 ± 418.5	-
				Control	8	508.8 ± 409.7	-
				Warm	11	289.5 ± 145.1	-
Atlantic cod	1	Acclimation (no bleeding)	CT_max_	Cold	6	168.5 ± 42.1	23.6 ± 2.4
				Control	6	168.6 ± 65.2	23.9 ± 2.8
				Warm	6	197.5 ± 70.3	24.5 ± 3.4
Goldsinny wrasse	2	Immersion	CT_max_	Cold	24	11.9 ± 4.4	8.4 ± 0.9
				Control	23	13.6 ± 5.0	8.6 ± 1.0
				Warm	24	13.1 ± 5.8	8.6 ± 1.1

Values are mean ± s.d.

Goldsinny wrasse (of unknown sex, age and life stage) were collected near the research station in the Gullmar Fjord on 11–13 June 2022, using baited traps (mesh lobster traps) deployed for 1 h. Wrasse were transported in buckets to the research station and held in a ~ 306 L tank (80 × 75 × 51 cm [L × W × H]) with black goby (*Gobius niger*), and a ~ 1350 L tank (275 × 79 × 62 cm [L × W × H]) with black goby and corkwing wrasse (*Symphodus melops*), neither of which were used in this study. Wrasses were fed mysid shrimp once daily to satiation.

For both cod and wrasse, plastic pipes and seaweed were provided to the tanks for shelter and the photoperiod was set to 18:6 h light:dark (5:00–23:00) to mimic natural conditions. Additional room lighting was manually switched on at approximately 8:00 and off at 22:00. The wrasse holding room also received natural light from windows. Individuals from both species started feeding within a day after introduction to their holding tanks. All tanks were supplied with flow-through, filtered and aerated seawater. The temperature of this water supply followed natural conditions in the area (cod: mean ± s.d. 14.83 ± 0.46°C, 12–21 June 2022; wrasse: mean ± s.d. 14.77 ± 0.20°C, 11–14 June 2022; data collected from the research station monitoring system, surface temperature, at http://www.weather.loven.gu.se/kristineberg/en/data.shtml). Furthermore, dissolved oxygen levels (DO_2_, % air saturation) and water quality parameters were measured in the cod tanks using a YSI ProSolo meter (Xylem Inc., USA) and a colorimetric test (JBL, Germany), respectively. DO_2_ ranged from 97.0 to 99.6% air saturation, and pH was 7.6 ± 0.0 with nitrate and nitrite levels < 0 mg L^−1^ in all cod tanks.

### Experiment 1

#### Cod transfusion treatment

The cod transfusion experiment was conducted on 21 June–3 July 2022. Cod were instrumented with intraperitoneal (i.p.) catheters to allow for repeated plasma injections into the i.p. cavity. Cod were initially anaesthetized by immersion in seawater containing 0.1 g L^−1^ MS-222 (tricaine methanesulfonate, Western Chemical, Canada) until ventilatory movements slowed down. After mass and standard length were recorded (Table 1), the cod were transferred to a surgery table on which the gills were continuously irrigated with chilled (4°C) and aerated seawater containing a maintenance dose of MS-222 (0.05 g L^−1^). An 18 G needle was used to puncture the body wall on the ventral side (anterior to the anus) to access the i.p. cavity. Subsequently, 2 F polyurethane tubing (Putnam Plastics Company, USA) filled with 0.9% saline containing 100 IU ml^−1^ heparin (LEO Pharma, Norway) was inserted into the i.p. cavity. The catheter proximal end was sealed with a 22 G PinPort™ (PNP3F22, Instech Laboratories, USA) and a PinPort Injector (PNP3M-F22, Instech Laboratories, USA) was used for injections. Correct cannula placement was confirmed by injecting a small amount (~0.1 ml) of heparinized saline and ensuring minimal pressure and leakage. The cannula was then secured to the skin with superglue at the insertion site, and with 4–0 silk sutures at two sites: posterior to the pectoral fin and next to the dorsal fin. The procedure was completed in 5 to 10 min. After recovery, cannulated cod were placed individually into cylindrical holding chambers, which were submerged in a tank supplied with 16°C aerated flow-through seawater. The holding chambers prevented the cod from placing heavy strains on/pulling out the catheter, and retrieval of catheter ends was facilitated with buoys. Three different chamber sizes were used to accommodate different cod sizes, with inner diameter×length dimensions of 7.5 × 35, 8.5 × 36 and 11 × 35 cm.

Catheter-instrumented cod (*n* = 17) were subjected to a transfusion treatment by injecting plasma from 8°C (cold), 16°C (control) or 21°C (warm) acclimating donor cod. For each injection, the plasma volume was 200 μl 100 g^−1^ of body mass. Injections were given 2–3 times per day at regular intervals (morning, midday, afternoon), with cod receiving 10 injections over the course of 4 days, except for one cod injected 10 times over 5 days, two cod injected 8 times over 4 days, and one cod injected 5 times over 2 days (the latter due to a catheter blockage). There were no mortalities associated with the catheter placement or the injections. Furthermore, 7 out of 17 of the cod continued feeding while housed in the chambers despite the confinement, whereby feeding cod were similarly distributed across the treatment groups. Atlantic cod also appear to cope well with short periods of fasting (<2 w) based on growth trials (Hanssen *et al.,* 2012). Altogether, Atlantic cod is an appropriate experimental species to undergo the transfusion treatment, given its relatively large body size facilitates blood withdrawal and catheter instrumentation, and it is tolerant to confinement in holding chambers during the treatment period.

#### Donor cod

Donor cod (*n* = 46) were transferred to acclimation tanks 24 h prior to blood collection. The cold and control acclimation tanks were at 8 and 16°C at the time of transfer. The temperature of the warm acclimation tank was gradually raised over 2 h from 16 to 21°C to avoid lethal heat stress in the cod. Warm acclimation temperatures above 21°C were tested but excluded from the experiment due to mortality amongst the donors. Acclimation tank temperatures were controlled by a thermostat (ITC-308-WIFI, Inkbird, China) and additionally monitored with a digital thermometer (testo-112, Testo, Germany). The 24 h acclimation period for donor cod was chosen to represent a potential ‘peak’ of hormonal changes during thermal acclimation. Although little is known about the temporal pattern of any endocrine regulation on thermal acclimation in fishes, we anticipated relatively rapid shifts, given that 24 h is already sufficient to induce increases in CT_max_ in warm acclimating fish (Healy and Schulte, 2012; Stewart *et al.,* 2023; De Bonville *et al.,* 2025). We are aware that acclimation periods beyond 24 h are usually needed to achieve full acclimation of CT_max_, especially in the case of cold acclimation, which has slower response dynamics compared to warm acclimation (Healy and Schulte, 2012). However, the aim of this study was to capture haematological signalling and physiological changes, which may occur early in the acclimation response (e.g. increased HSP transcript and protein levels in < 24 h; Lewis *et al.,* 2016), rather than when the plateau in CT_max_ is reached.

After acclimation, we collected blood from a subset of the donor cod (*n* = 28) to obtain plasma for transfusion into recipient cod. These cod were initially anaesthetized with 0.1 g L^−1^ MS-222 (24 out of 28 cod) or with 0.04 ml L^−1^ clove oil (C-8392, Sigma, USA) in seawater (4 out of 28 cod), and then weighed (Table 1). While gills were irrigated with a maintenance dose of 0.05 g L^−1^ MS-222 in seawater, blood was quickly drawn by caudal puncture with heparinized syringes (100 IU ml^−1^ in 0.9% saline), and centrifuged for 1 min at 5000 rpm in a refrigerated centrifuge (5804 R, Eppendorf) at 4°C. The plasma was aliquoted into 1.5 ml Eppendorf tubes kept on ice, and either used immediately for injections of recipient cod whenever possible, or stored at −80°C until future use (thawed on ice beforehand). Thawed plasma was never refrozen to avoid repeated freeze–thaw cycles, and discarded at the end of the injection event if not used. Under these storage conditions, many hormones (e.g. TH, glucocorticoids, sex steroids) in Atlantic cod plasma remain stable with minimal degradation (Kjesbu *et al.,* 1996; Cyr *et al.,* 1998; Comeau *et al.,* 2000; Norberg *et al.,* 2004). Several plasma samples were set aside and stored at −80°C for the measurement of T_3_ levels. Donor cod that were bled were euthanized by spinal cord severance.

From the remainder of the donor cod (*n* = 18), no blood was collected; instead, these cod were tested for CT_max_ following the 24 h acclimation period.

#### Cod respirometry

SMR were estimated by measuring mass-specific oxygen uptake rates (*Ṁ*O_2_) of the transfusion recipient cod (*n* = 17) fasted one day (≥24 h) prior to respirometry. Three intermittent-flow respirometers were custom designed using two cylindrical Plexiglas chambers (large: 3.07 L, small: 1.26 L) and one plastic box chamber (5.05 L) (Table S1). Respirometry chambers were submerged in a seawater bath kept at 16°C, and temperature was recorded continuously using a logger (TC-08, Pico Technologies, UK) and a temperature sensor (FireSting-O_2_, PyroScience, Germany). A recirculation pump connected to each respirometry chamber ensured that water was well mixed throughout each *Ṁ*O_2_ measurement. DO_2_ was measured inside each chamber using a fibre-optic oxygen sensor (FireSting-O_2_, PyroScience, Germany), and a flushing pump was intermittently activated to ensure DO_2_ did not drop below 80% air saturation.

After receiving the last plasma injection, cod were transferred from the holding chambers to respirometers and given a minimum of 4 h (>8 h for 14 out of 17 cod) to adjust to confinement conditions before *Ṁ*O_2_ measurements began (Chabot *et al.,* 2016). After sealing the respirometers, DO_2_ was measured every 2 s (0.5 Hz sampling frequency). Cycle duration (flushing and recirculation periods) was adjusted according to the size of the fish and respirometer (Table S1). The *Ṁ*O_2_ measurements were conducted overnight over a 12–18 h time period, and respirometry trials were completed within 8 days. One cod injected with plasma from cold-acclimating donors died overnight in the respirometry chamber because of a flush pump failure, before SMR could be estimated.

*Ṁ*O_2_ (mg O_2_ h^−1^ g^−1^) was calculated as:


(1) \begin{align*} {\dot{M}\mathrm{O}}_2=\frac{\left(\ {V}_r-{V}_f\right)\times \varDelta Cw{O}_2}{\varDelta t\times{M}_f} \end{align*}

where *V*_r_ and *V*_f_ are the volume of the respirometer and fish, respectively, (1 g of fish corresponds to ~ 1 ml), Δ*C*wO_2_ is the change in oxygen concentration (mg L^−1^) in the respirometer water, Δ*t* is the time over which Δ*C*wO_2_ is measured, and *M*_f_ is the fish mass (g) (Clark *et al.,* 2013). Any *Ṁ*O_2_ measurements with a *R*^2^ < 0.90 for the linear slope were removed from the dataset (5 measurements due to a fish, which likely struggled/activity may have changed during the recirculation period, 4 measurements due to an incomplete cycle at the end of the *Ṁ*O_2_ measurements and 36 measurements due to a flush pump failing to turn off causing a skipped cycle). After removing the fish from the respirometers at the end of each trial, background measurements were conducted for at least 20 min to account for microbial respiration and algal photosynthesis, and subtracted from the SMR of the respective fish. The fish were then transferred to the CT_max_ arena to be successively tested. All *Ṁ*O_2_ data were analyzed using the *respR* package in R (Harianto *et al.,* 2019), whereby SMR was calculated as the average of the lowest 10% of the rates. Full details of the respirometry setup and data analysis can be found in Table S2 following reporting guidelines by Killen *et al.* (2021).

#### Donor cod plasma T_3_ levels

Total triiodothyronine (T_3_) levels in donor cod plasma were measured using a commercial competitive radioimmunoassay (RIA) kit (KIP1631, DiaSource, Belgium) based on ^125^I-labelled T_3_ tracer (111 kBq). Samples were stored for four months at −80°C followed by one year at −20°C prior to analysis. Given that the RIA kit was designed for human serum, assay compatibility with cod plasma was first verified using sample pools from each donor cod treatment. Samples were thawed on ice, and approximately half of the samples were run in triplicate and the remainder in duplicate, while standards and controls were run in duplicate. Sample T_3_ concentrations were calculated from the standard curve, which was included in each assay along with at least one control sample (supplied with kit) and an internal control from pooled cod plasma. Assays also included a total count and non-specific binding blank. The lowest concentration detectable was 0.22 nmol ml^−1^. Intra- and inter-assay coefficients of variation (CV) were 3.9% (*n* = 12 duplicates/triplicates) and 3.5% (*n* = 5 duplicates), respectively. One sample with a CV > 15% was reanalyzed. Four samples were diluted 1.4× using the zero calibrator (human serum containing 0 nmol L^−1^ T_3_) of which two samples still slightly exceeded the standard curve range (0–9.67 nmol L^−1^; 0–6.30 ng ml ^− 1^) after dilution. For these two samples, T_3_ levels were estimated as the highest standard concentration. Radioactivity was measured using a gamma counter (Cobra Auto Gamma, model 5003, Packard Instrument Company, USA).

### Experiment 2

#### Wrasse immersion treatment

The wrasse immersion experiment was conducted on 14–21 June 2022. Groups of 12 wrasse were randomly distributed across six plastic crates (Bauhaus 10 753 254, Germany; 40 × 30 × 22 cm [L × W × H]) lined with fine nylon mesh, with five plastic pipes and artificial plastic seaweed for shelter. These crates were submerged in a 360-L tank supplied with 16°C flow-through, aerated seawater. Crates were each assigned to one of three immersion treatments (two crates per treatment): 8°C (cold), 16°C (control) or 24°C (warm). After being placed in their crates, wrasse were fed mysid shrimp once more to satiation and were then fasted for the remainder of the experiment to prevent water quality deterioration.

**Figure 1:**
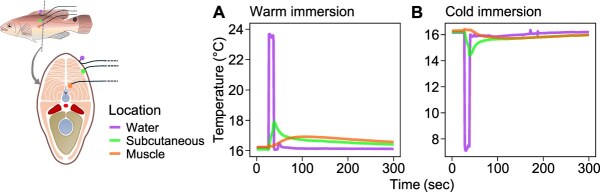
Temperature changes during rapid immersion in warm and cold seawater in skin and muscle in goldsinny wrasse (*Ctenolabrus rupestris*). A wrasse (24.9 g) acclimated to 16°C was anaesthetized and immersed for 10 s in (A) 24°C (warm) or (B) 8°C (cold) seawater, and subsequently returned to 16°C seawater. Temperatures were recorded with thermocouples on the outer surface of the skin (water, purple), directly under the skin surface (subcutaneous, green), and in deep muscle (muscle, orange) in the time period before, during and after immersion.

Before beginning the immersion treatments, the extent of temperature change at different body depths during a 10 s immersion was verified on one 16°C-acclimated wrasse (24.9 g) anaesthetized in 0.1 g L^−1^ MS-222. Thermocouple probes (Type K, SE029, Pico Technologies, UK) connected to a data logger (TC-08) were attached to the outside of the skin, inserted under the skin surface (subcutaneous) and into deep muscle (dorsal side). Next, 10 s test immersions in 8°C (cold) and 24°C (warm) water were conducted. The fish was transferred back into 16°C (holding temperature) water in between immersions. Subcutaneous temperature change was limited to < 1.9°C, and deep muscle temperature change was < 0.7°C during both immersions (Fig. 1). This confirmed that only the outer surface of the skin equilibrated with the water temperature during the immersions.

The immersion treatments started the day after wrasse were transferred into the crates. Five times daily over five days, crates with wrasse from each treatment group were removed from the holding tank and immersed for 10 s in a water bath containing either cold, control or warm water, before being returned to the holding tank. During the immersion process, wrasse were minimally exposed to air (≤1 s) as water baths were placed directly next to the holding tank, so that crates could be quickly transferred. Immersions were done at the same time of day (at 7:30, 10:30, 13:30, 16:30 and 19:30) at 3 h intervals throughout the treatment period (Table S3). The order of crate immersion and position of crates in the holding tank were changed after each round of immersions. Temperature was measured with a high-accuracy digital thermometer (testo-112, Testo, Germany) in the holding tank and water baths before and after the immersions (Table S3). The immersion treatment frequency (five times daily) and period (over five days) were chosen based on prior studies about CT_max_ acclimation dynamics (Healy and Schulte, 2012; Stewart *et al.,* 2023; De Bonville *et al.,* 2025) and repeated intermittent heating (Loeb and Wasteneys, 1912) in fishes, and were estimated to allow for a clear thermal signal over a sufficiently long exposure time. Furthermore, goldsinny wrasse were well suited as the experimental species to undergo the immersion treatment, given that it readily acclimatizes to laboratory conditions (Sundin and Jutfelt, 2016), is not highly sensitive to netting stress, and possesses a robust skin. Indeed, no skin abrasions or wounds were observed amongst the fish during the treatment period. One fish died in the control treatment due to confinement of the fish between the crate and the mesh lining the crate.

### CT_max_ tests

Methods for all CT_max_ tests followed the procedures described by Morgan *et al.* (2018) for zebrafish, with some modifications.

Cod CT_max_ tests were conducted within 1 h following overnight respirometry (recipient cod) or thermal acclimation (donor cod), and with 1–3 individuals at a time. Given the larger body size of cod compared to zebrafish, a slower ramping rate of 0.1°C min^−1^ (actual rate 0.1 ± 0.0°C min^−1^, mean ± s.d.) was used. The CT_max_ setup consisted of a rectangular box (‘arena’, 56 × 36 × 28 cm [L × W × H]) of opaque plastic with a transparent lid, filled with 30 L of aerated 16°C seawater. Mixing of water inside the arena to ensure thermal homogeneity was achieved with a pump (EHEIM Universal 300, Germany) attached to the outside of the box, and the water was heated using 100 W titanium heaters (Aqua Medic, Germany) and 200 W glass heaters (EHEIM).

Wrasse CT_max_ tests were conducted the day after the last immersions between 9:00 and 17:00. All individuals (*n* = 11–12) from each treatment group were tested together, with groups tested in a balanced order. Trials were conducted in a large styrofoam tank (‘arena’, 50 × 32 × 32 cm [L × W × H], 17.5 cm water depth) connected to a sump used for heating (37 × 37 × 34.5 cm [L × W × H], 6.5 cm water depth) (total volume of 35 L). Two titanium heating rods (500 W and 300 W, Aqua Medic, Germany) were placed in the sump to reach a 0.3°C min^−1^ ramping rate during trials (actual rate 0.3 ± 0.0°C min^−1^, mean ± s.d.). Three water pumps (EHEIM Universal 300, Germany) were placed in the sump to ensure homogeneous mixing between the arena and the heating sump: two pumps transferred the heated water to the CT_max_ arena containing the fish, while the third pumped water from the arena back to the sump via an outlet in the bottom (sealed with mesh). Wrasses were habituated at their acclimation temperature (16.3 ± 0.2°C, mean ± s.d.) for 5 min prior to heating.

For both species, a high-precision digital thermometer (testo-112, Testo, Germany, accuracy: ±0.2°C, resolution: 0.1°C) was placed in the CT_max_ arena to continuously monitor water temperature and confirm a steady heating rate. The temperature at loss of equilibrium (LOE), defined as a loss of upright body position for more than 3 s, was used as the endpoint for CT_max_. When an individual reached LOE, it was immediately transferred to aerated 16°C seawater for recovery. Once survival was confirmed after 30 min, fish were euthanized using 0.2 g L^−1^ MS-222 or 0.04 ml L^−1^ clove oil, and mass and standard length were recorded (Table 1). All cod and wrasse survived the CT_max_ trials, except for two cold-acclimated cod, which died within 30 min after reaching their CT_max_.

### Data analysis and statistics

Statistical analyses were performed in R v4.4.1 (R Core Team, 2024), using RStudio v2024.04.2 (Posit team, 2024). Data were visualized with *ggplot2* (Wickham and Wickham, 2016). All response variables were analyzed with one-way ANOVAs (aov function, *stats* package) to assess the effect of the transfusion, thermal acclimation or immersion treatments. In case of a significant effect, differences between individual groups were analyzed using Tukey’s HSD post hoc comparisons (PostHocTest function, *DescTools* package; Signorell *et al.,* 2022). All model fits were graphically assessed for violations of the assumptions for normality and homogeneity using quantile quantile and residual-fit plots, respectively. Data are reported as the mean ± standard error (s.e.m.) unless specified otherwise. The level of significance was set at α = 0.05.

## Results

### Experiment 1

#### Cod CT_max_

**Figure 2:**
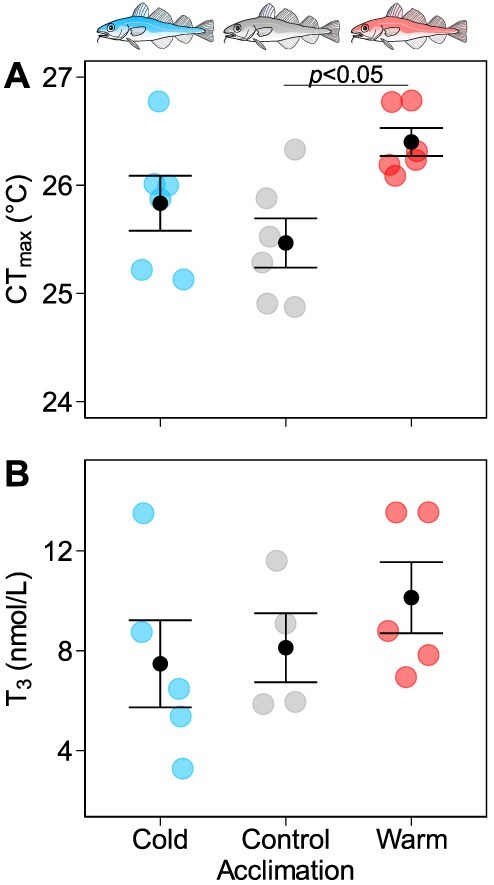
The effect of 24 h thermal acclimation on CT_max_ and plasma T_3_ levels in Atlantic cod (*Gadus morhua*) donors. (A) CT_max_ (°C) was measured in cod after 24 h following transfer from 16 to 8°C (cold), 16°C (control) or 21°C (warm) temperatures (*n* = 6 per acclimation group). The same acclimation treatment was used for donor cod prior to blood collection, to obtain plasma for transfusion into recipient cod. (B) Plasma T_3_ levels in donor cod acclimating to 8°C (cold) (*n* = 5), 16°C (control) (*n* = 4) or 21°C (warm) (*n* = 5) temperatures for 24 h. Coloured data points represent data for individual fish (blue: cold, grey: control, red: warm acclimation group), and black data points with error bars represent the mean ± s.e.m. for each acclimation group. Acclimation groups that are statistically significant (α = 0.05) according to Tukey’s post hoc comparisons are marked with a horizontal bar and *P* value.

There was a significant effect of acclimation treatment on CT_max_ in cod (*F*_2,15_ = 4.992, *P* = 0.022; Fig. 2A). Warm-acclimated cod had a higher CT_max_ compared to controls (26.4 ± 0.1°C *v.* 25.5 ± 0.2°C, *P* = 0.018). The CT_max_ of the cold-acclimated cod (25.8 ± 0.3°C) did not significantly differ from either the control or warm-acclimated cod (*P* = 0.454 and 0.172, respectively).

**Figure 3:**
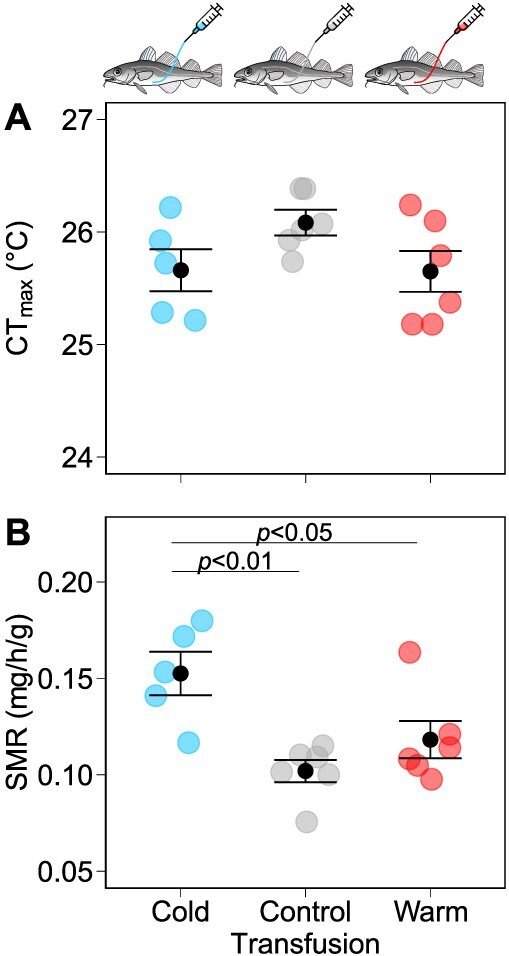
CT_max_ and SMR in transfusion-treated Atlantic cod. (A) CT_max_ (°C) was measured in recipient cod directly following overnight respirometry to determine (B) mass-specific SMR (mg O_2_ h^−1^ g^−1^). Recipient cod was injected with plasma from 8°C (cold), 16°C (control) or 21°C (warm) acclimating donors (*n* = 6 per transfusion treatment except *n* = 5 for cold plasma recipients). Coloured data points represent data for individual fish (blue: cold, grey: control, red: warm plasma transfusion treatment), and black data points with error bars represent the mean ± s.e.m. for each transfusion treatment. Transfusion treatment groups that are statistically significant (α = 0.05) according to Tukey’s post hoc comparisons are marked with a horizontal bar and *P* value.

Transfusion of donor plasma from warm- or cold-acclimated fish into controls did not affect CT_max_ in the recipients (*F*_2,14_ = 2.409, *P* = 0.126; Fig. 3A). Cod that were injected with plasma from donors acclimated to cold, control and warm temperatures had a CT_max_ of 25.7 ± 0.2°C, 26.1 ± 0.1°C and 25.7 ± 0.2°C, respectively.

The overall CT_max_ of acclimated cod did not significantly differ from transfused cod (*F*_1,33_ = 0.271, *P* = 0.606; Figs 2A and 3A) although control-acclimated cod had a lower CT_max_ than cod injected with plasma from control-acclimated donors (25.5 ± 0.2°C *v.* 26.1 ± 0.1°C, *P* = 0.036). This indicates that catheterization combined with the confinement during transfusion and respirometry had no detrimental effect on cod CT_max_.

#### Recipient cod SMR

The plasma transfusion treatment did affect SMR in cod (*F*_2,14_ = 7.877, *P* = 0.005; Fig. 3B). Cod given plasma injections from cold-acclimated donors had a 1.5-fold higher SMR than did cod injected with control plasma (0.153 ± 0.011 mg O_2_ h^−1^ g^−1^  *v.* 0.102 ± 0.006 mg O_2_ h^−1^ g^−1^, *P* = 0.004) and also had a slightly elevated SMR compared to cod receiving plasma transfusions from warm-acclimated donors (0.118 ± 0.010 mg O_2_ h^−1^ g^−1^, *P* = 0.046).

#### Donor cod plasma T_3_ levels

The plasma T_3_ levels did not significantly differ amongst the donor groups (*F*_2,11_ = 0.834, *P* = 0.46). Total T_3_ levels in donor cod plasma ranged from 3.28 to 13.54 nmol L^−1^ (2.13–8.81 ng ml^−1^; Fig. 2B).

### Experiment 2

#### Wrasse CT_max_

**Figure 4:**
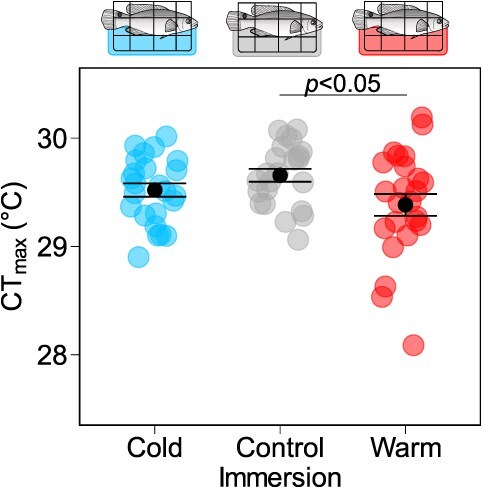
Acute warming tolerance of immersion treated goldsinny wrasse. CT_max_ (°C) was measured in 16°C-acclimated wrasse that underwent brief and frequent immersions in 8°C (cold) (*n* = 24), 16°C (control) (*n* = 23) or 24°C (warm) (*n* = 24) water. Coloured data points represent the CT_max_ of individual fish (blue: cold, grey: control, red: warm immersion treatment), and black data points with error bars represent the mean ± s.e.m. for each immersion treatment group. Immersion treatments that are statistically significant (α = 0.05) according to Tukey’s post hoc comparisons are marked with a horizontal bar and *P* value.

There was a weak effect of immersion treatment on CT_max_ in wrasse (*F*_2,68_ = 3.141, *P* = 0.0496) (Fig. 4). Wrasse immersed in warm water had on average a lower CT_max_ than did controls (mean ± s.e.m., 29.4 ± 0.1°C *v.* 29.7 ± 0.1°C, *P* = 0.0383). This difference appears to be mainly driven by one fish in the warm immersion treatment, however, which had a relatively low CT_max_ (28.1°C) resulting in higher variation in the warm immersion group compared to controls (s.d., 0.5°C *v.* 0.3°C). Indeed, upon removal of this datapoint, immersion treatment no longer had an effect on CT_max_ (*F*_2,67_ = 2.392, *P* = 0.099). No difference in CT_max_ was detected between the cold immersed wrasse (29.5 ± 0.1°C) and the control and warm immersion treatment groups (*P* = 0.431 and 0.414, respectively).

## Discussion

In this study, we investigated some potential mechanisms of neural and endocrine control of thermal acclimation in fish. As the roles of thermoreception and hormones in this process have remained elusive, we used transfusions of plasma extracted from thermally acclimating Atlantic cod (Experiment 1) and transient cold/warm water bath immersions of goldsinny wrasse (Experiment 2) as the experimental treatments. We found no evidence for a warm acclimation response in cod receiving plasma from warm-acclimating donors. In cod receiving plasma from cold-acclimating donors, we did see a 50% increase in SMR compared to controls. The increase in SMR after transfusion with cold-acclimated donor plasma is consistent with the increase in metabolic rates, enzyme activities and tissue mitochondrial volume density and respiration capacity often observed in cold-acclimated fishes and other ectotherms (Johnston and Dunn, 1987; Dhillon and Schulte, 2011; Seebacher *et al.,* 2015; Cominassi *et al.,* 2022). The postulated benefit of upregulating the metabolism at cold temperatures is to compensate for the direct physicochemical effects of cooling on enzymatic function and biological rates in general, allowing the animal to maintain a more stable performance across temperatures (Angilletta, 2009; Schulte, 2015; Jutfelt *et al.,* 2024; Schulte, 2024). As such, our findings provide preliminary evidence in favour of endocrine mediation of cold acclimation in Atlantic cod. Our study demonstrates the value of plasma transfusions as a useful first step in testing for the involvement of circulating hormones and other plasma-borne molecules in regulating an animal’s physiological process, as showcased by Enok *et al.* (2012). The next step would be to identify the specific plasma mediators involved.

Given the putative role of TH as an endocrine regulator of thermal acclimation in ectotherms (Hoar and Eales, 1963; Prosser *et al.,* 1991; Little *et al.,* 2013; Little and Seebacher, 2014; Zak *et al.,* 2017; Zak and Manzon, 2019; Le Roy and Seebacher, 2020; Little, 2021), including possibly upregulating the metabolism during cold acclimation in fishes (Little *et al.,* 2013; Little and Seebacher, 2014; Little, 2021), we measured plasma T_3_ (3,5,3′-T_3_) levels in the donor cod. The plasma T_3_ levels (2–9 ng ml^−1^) were within the range reported for this species in the literature (Cyr *et al.,* 1998; Comeau *et al.,* 2000). Although serum T_3_ levels of cod can correlate negatively with water temperature (with highest levels during mid-winter) (Comeau *et al.,* 2000), plasma T_3_ levels measured here did not differ amongst donor cod acclimation groups. These findings are in line with Cyr *et al.* (1998) who also found no change in plasma T_3_ levels in response to thermal acclimation in cod. Considering the lack of a clear link between T_3_ levels in donor plasma and SMR in recipients, and that T_3_ is the main bioactive form of TH (Little *et al.,* 2013; Deal and Volkoff, 2020), we did not find evidence that TH regulation explains the metabolic effects of plasma transfusion we observed. Early work by Klicka (1965) in goldfish (*Carassius auratus*) also failed to link thyroid gland activity with metabolic changes during thermal acclimation. However, future studies could explore the involvement of other aspects of the TH pathway/axis in the thermal acclimation process of fishes, including thyroid-stimulating hormone (TSH), T_2_ and T_4_ plasma levels, TH receptor dynamics (spatiotemporal changes in density, sensitivity and isoforms), the proportion of unbound T_3_ amongst total T_3_ levels, tissue deiodinase enzyme activities and feedback mechanisms. Furthermore, the role of other hormones could be explored, such as that of glucocorticoid (GC) hormones (cortisol and corticosterone), which have been shown to be important temperature-dependent physiological regulators of the metabolism in other ectothermic vertebrates (amphibians and reptiles) (Jessop *et al.,* 2018; Holden *et al.,* 2021). In fish, cortisol and corticosterone are primarily known as stress hormones (Xiao *et al.,* 2021) but are also associated with acute upper thermal tolerance (CT_max_) and the cold/heat shock response (LeBlanc *et al.,* 2012; Shaughnessy and McCormick, 2018; Reid *et al.,* 2022). In addition to hormones, there may be other plasma metabolites, such as glucose, fatty acids and amino acids that are involved in metabolic compensation during cold acclimation in fishes, which could be further explored (Reid *et al.,* 2022; Wang *et al.,* 2024).

Acute upper thermal tolerance as assessed by CT_max_ is highly responsive to thermal acclimation (Fry, 1947; Morgan *et al.,* 2022; Jutfelt *et al.,* 2024; Ruthsatz *et al.,* 2024). Therefore, it is surprising that plasma transfusion appears to have successfully transferred cold acclimation of metabolism but not thermal tolerance. Meanwhile, the 24 h warm acclimation did increase CT_max_ by 0.9°C in the donor cod. These findings suggest that the mechanisms behind thermal acclimation of metabolic and thermal tolerance traits are distinct in fishes. It may be that only some components of thermal acclimation such as metabolism are regulated by circulating hormones. It is also possible that certain hormonal factors in donor plasma were at a too low concentration or were degraded after injection, before these could have an effect on thermal tolerance. We infused recipients with donor plasma via intraperitoneal (i.p.) cannulas to accommodate frequent multiday injections, but intravenous (i.v.) transfusions might allow for more pronounced hormonal effects given the direct delivery to the blood circulation. However, the management of cannula retention and blood clotting may be difficult with repeated daily i.v. transfusions. Furthermore, the i.p. injection route of administration for hormones has been proven effective based on prior studies (Lam, 1982; Schmid *et al.,* 2003; Shved *et al.,* 2011). Alternatively, we cannot rule out the possibility that the observed metabolic effect of plasma transfusion in this study is a spurious result due to the low number of fish tested. Therefore, we encourage additional investigations into the physiological mechanisms and responses of thermal acclimation, using more robust sample sizes across a range of species.

Contrary to our prediction, the immersion treatment did not trigger thermal acclimation in goldsinny wrasse, based on CT_max_. Our CT_max_ results were similar to those recently reported in another study for this species (De Bonville *et al.,* 2025). We, therefore, conclude that cutaneous thermosensation is unlikely to be a main controller of thermal acclimation, or that traits other than thermal tolerance were affected. However, the immersion protocol with five daily exposures for five days (with a cumulative exposure time of 4 min) may have been insufficient to stimulate central pathways controlling thermal acclimation. Whether longer durations of each immersion and/or the overall treatment period (e.g. 1 h long exposures for over a week, as in Loeb and Wasteneys, 1912) would induce an observable effect on thermal tolerance remains to be tested. Fish have thermosensitive neurons in the skin, but also in the visceral organs, retina, pineal gland and the brain, whereby the brain integrates the thermosensory information from the peripheral tissues (Gracheva and Bagriantsev, 2015; Haesemeyer, 2020; Nisembaum *et al.,* 2022). For the onset of thermal acclimation, it might be a prerequisite that the thermosensory neurons in deeper tissues, including the brain itself, are stimulated by the temperature change. The downside of longer immersions is that it becomes difficult to disentangle peripheral sensory detection of external temperature from direct thermal effects on somatic cells.

## Conclusion

Using a set of experiments in cod and wrasse, we found some evidence for endocrine control of metabolic compensation during cold acclimation, but no evidence for any acclimation effect from brief cutaneous exposures to temperature changes. Altogether, our results and approach provide several opportunities for further research avenues with relevance both in fishes and other ectotherms, such as efforts to identify the specific hormonal or plasma-borne factors regulating thermal acclimation. For most ectotherms, thermal acclimation is an essential coping strategy (Seebacher *et al.,* 2015; Somero *et al.,* 2016). A better understanding of the neural and endocrine control underlying this process might facilitate predictions of the impacts from environmental perturbations, including those from climate change, on this group of animals. For instance, this fundamental knowledge can be useful when projecting, which fish species, assemblages, and communities may be vulnerable to marine cold-spells and heatwaves, and which may be able to cope with these events by improving performance and survival through cold and warm acclimation, respectively (Somero, 2010; Johansen *et al.,* 2021; Harvey *et al.,* 2022). Furthermore, the information in this study about the physiology of thermal acclimation in Atlantic cod and goldsinny wrasse contributes to the conservation and management of these ecologically and socio-economically important species. This is because acclimation responses to thermal stress and their regulatory mechanisms are key physiological traits that ultimately could support forecasting and policy decision-making for both wild and captive populations of cod and wrasse.

## Supplementary Material

Web_Material_coaf042

## Data Availability

Data presented in this study are publicly available on Zenodo: https://doi.org/10.5281/zenodo.13823397.
