# Supplementary material for: Putative neural and endocrine control of thermal acclimation in fish
Source: Conserv Physiol. 2025 Jun 17;13(1):coaf042. doi: 10.1093/conphys/coaf042 (PMC12204395; doi:10.1093/conphys/coaf042)
Supplement: Web_Material_coaf042 [file web_material_coaf042.zip › Leeuwis_et_al_2025_Supplement .pdf]

Supplementary materials for

Putative neural and endocrine control of thermal acclimation in fish

Robine H.J. Leeuwis*, Rachael Morgan, Anna H. Andreassen, Lorena Silva-Garay, Zara-Louise Cowan, Eirik R. Åsheim, Jeremy De Bonville, Sandra A. Binning, Graham D. Raby, Fredrik Jutfelt

***Author for correspondence:** rhjleeuwis@gmail.com

This PDF file includes:

Supplementary tables 1 to 3

Table S1: Details on the intermittent-flow respirometry trials with plasma-injected cod (*Gadus morhua*).

Date	Fish			Respirometer		$\dot{M}O_2$ measurement time			Cycle duration (sec)		Cycles
(day-month-year)	Treatment	ID	Mass (g)	Volume (L)	Type	Start	End	Total (h)	Flushing	Recirculation	(nr.)
25-06-2022	Warm	3	226	3.07	Large	12:45 AM	3:00 PM	14.8	210	240	114
	Cold	4	143	1.26	Small				150	90	213
26-06-2022	Cold	1	200	3.07	Large	11:30 PM	11:30 AM	12	210	300	78
	Warm	5	214	5.05	Box				210	360	70
27-06-2022	Control	6	223	3.07	Large	7:30 PM	10:45 AM	15.3	210	360	96
	Control	7	126	5.05	Box				240	420	82
	Warm	9	89	1.26	Small				180	270	121
28-06-2022	Cold	8	157	3.07	Large	4:30 PM	10:30 AM	18	210	300	127
29-06-2022	Control	10	242	5.05	Box	8:00 PM	9:45 AM	13.8	240	360	82
	Cold	11	205	3.07	Large				120	330	109
30-06-2022	Warm	12	189	3.07	Large	11:00 PM	10:00 AM	11	120	420	73
	Warm	13	123	5.05	Box				210	570	49
	Cold	14	119	1.26	Small	10:30 PM	10:00 AM	11.5	150	270	95
01-07-2022	Control	15	194	3.07	Large	11:00 PM	10:30 AM	11.5	120	300	95
	Control	16	141	5.05	Box	10:30 PM	10:30 AM	12	150	270	102
02-07-2022	Warm	18	263	3.07	Large	7:30 PM	9:30 AM	14	120	240	141
	Control	19	146	5.05	Box	6:00 PM	9:30 AM	15.5	120	270	144

Table S2: Checklist of criteria from Killen et al. (2021) to be reported when using aquatic intermittent-flow respirometry to estimate SMR.

Number	Criterion and category	Description
**Equipment, materials and setup**		
1	Body mass of animals at time of respirometry	Mean±s.d. 176.5±49.8 g
2	Volume of empty respirometers	5.05 L (box chamber), 3.07 L (large chamber), and 1.26 L (small chamber)
3	How chamber mixing was achieved	Using a recirculating loop (inside 5.05 L chamber, and outside 3.07 and 1.26 L chambers)
4	Ratio of net respirometer volume (plus any associated tubing in mixing circuit) to animal body mass	Mean±s.d. 59.7±25.4 g/L
5	Material of tubing used in any mixing circuit	Green vinyl tubing (4005943, EHEIM, Germany)
6	Volume of tubing in any mixing circuit	Not recorded
7	Confirm volume of tubing in any mixing circuit was included in calculations of oxygen uptake rates	Volume of recirculating tubing is included in the volume of empty chambers (3.07 and 1.26 L chambers)
8	Material of respirometer	Plastic (5.05 L chamber) and Plexiglas (3.07 and 1.26 L chambers)
9	Type of oxygen probe and data recording	Fiber-optic oxygen sensor (FireSting-O_2, PyroScience, Germany)
10	Sampling frequency of water dissolved oxygen	0.5 Hz
11	Placement of oxygen probe	Oxygen probe was placed in the mixing circuit
12	Flow rate during flushing and recirculation, or confirm that chamber returned to normoxia during flushing	Flow rate of flushing and recirculation loops was 300 L/h, and all chambers returned to normoxia during flushing
13	Timing of flush/closed cycles	Cycle duration was adjusted for each fish and respirometer (see Table S1 for details)
14	Wait (delay) time excluded from closed measurement cycles	No wait time was required to obtain a R^2 of >0.90 for measurements
15	Frequency and method of probe calibration (for both 0 and 100% calibrations)	Calibrated at 100% air saturation in seawater bubbled with air, and used factory calibration for 0% air saturation. Calibrated daily before each group of fish were measured
16	State whether software temperature compensation was used during recording of water oxygen concentration	Yes
**Measurement conditions**		
17	Temperature during respirometry	16°C
18	How temperature was controlled	Temperature was maintained at 16°C by mixing the surface (15–18°C) and deep (12–15°C) seawater supply of the research station, and was recorded continuously using a logger (TC-08, Pico Technologies, UK) and a temperature sensor

		(FireSting-O_2, PyroScience, Germany)
19	Photoperiod during respirometry	18 L:6 D (5:00-23:00) with additional room lighting at 8:00-22:00
20	If (and how) ambient water bath was cleaned and aerated during measurement of oxygen uptake (e.g. filtration, periodic or continuous water changes)	Respirometers were inside the holding tank, which received flow-through, filtered, and aerated seawater. Feed remains were also regularly siphoned out to clean the tank bottom
21	Total volume of ambient water bath and any associated reservoirs	~1440 L
22	Minimum water oxygen dissolved oxygen reached during closed phases	80% air saturation
23	State whether chambers were visually shielded from external disturbance	Chambers were visually shielded from external disturbance by being covered up with opaque lids
24	How many animals were measured during a given respirometry trial (i.e. how many animals were in the same water bath)	1–3 cod were measured during each respirometry trial, and respirometers were inside the same holding tank
25	If multiple animals were measured simultaneously, state whether they were able to see each other during measurements	Fish were able to see each other during measurements
26	Duration of animal fasting before placement in respirometer	Minimum of 1 d
27	Duration of all trials combined (number of days to measure all animals in the study)	Respirometry trials were completed in 8 d (start date: 25-06-2022, end date: 03-08-2022)
28	Acclimation time to the laboratory (or time since capture for field studies) before respirometry measurements	Minimum of 10 d
**Background respiration**		
29	State whether background microbial respiration was measured and accounted for, and if so, method used (e.g. parallel measures with empty respirometer, measurements before and after for all chambers while empty, both)	Background respiration was measured after each trial and for all chambers while empty, and was accounted for in the calculation of SMR
30	If background respiration was measured at beginning and/or end, state how many slopes and for what duration	Background respiration was measured for a minimum of 20 min using a single slope
31	State how changes in background respiration were	No temporal changes in background respiration were modelled

	modelled over time (e.g. linear, exponential, parallel measures)	
32	Level of background respiration (e.g. as a percentage of SMR)	Background respiration was ~0.6% of SMR
33	State method and frequency of system cleaning (e.g. system bleached between each trial, UV lamp)	Respirometers were cleaned with dilute bleach before starting each trial
**Standard or routine metabolic rate**		
34	Acclimation time after transfer to chamber, or alternatively, time to reach beginning of metabolic rate measurements after introduction to chamber	A minimum of 4 h (>8 h for 14 out of 17 cod)
35	Time period, within a trial, over which oxygen uptake was measured (e.g. number of hours)	12–18 h
36	Value taken as SMR/RMR (e.g. quantile, mean of lowest 10 percent, mean of all values)	SMR was calculated as the mean of the lowest 10% of the rates
37	Total number of slopes measured and used to derive metabolic rate (e.g. how much data were used to calculate quantiles)	After acclimation, 49–213 slopes were measured per individual to derive metabolic rate (see Table S1 for details)
38	State whether any time periods were removed from calculations of SMR/RMR (e.g. data during acclimation, periods of high activity [e.g. daytime])	Data obtained during the acclimation time to chambers were not used for the SMR calculations
39	r^2 threshold for slopes used for SMR/RMR (or mean r^2)	0.90
40	Proportion of data removed due to being outliers below r^2 threshold	R^2 was <0.90 for 45 out of 1807 measurements (2%), which was primarily due to cycle issues (see Methods for details), and these outliers were removed from the dataset
**Maximum metabolic rate**		
41	When MMR was measured in relation to SMR (i.e. before or after)	n/a
42	Method used (e.g. critical swimming speed respirometry, swim to exhaustion in swim tunnel, or chase to exhaustion in tank or respirometer)	n/a
43	Value taken as MMR (e.g. the highest rate of oxygen uptake value after transfer, average of highest values)	n/a

44	Length of activity challenge used for estimating MMR (e.g. duration and water velocity increment of swim test, duration of chase in minutes or until exhaustion, etc.)	n/a
45	If MMR was measured post-exhaustion, state whether further air-exposure was added after exercise	n/a
46	If MMR was measured post-exhaustion, provide time until transfer to chamber after exhaustion and time to start of oxygen uptake recording	n/a
47	Duration of slopes used to calculate MMR (e.g. 1 min, 5 min, etc.)	n/a
48	Slope estimation method for MMR (e.g. rolling regression, sequential discrete time frames)	n/a
49	How absolute aerobic scope and/or factorial aerobic scope is calculated (i.e. using raw SMR and MMR, allometrically mass-adjusted SMR and MMR, or allometrically mass-adjusting aerobic scope itself)	n/a
**Data handling and statistics**		
50	Sample size	*n*=6 (control plasma recipients), 6 (warm plasma recipients), 5 (cold plasma recipients)
51	How oxygen uptake rates were calculated (software or script, equation, units, etc.)	Oxygen uptake rates (in mg O_2 h^{-1} g^{-1}) were calculated according to the equation by Clark et al. (2013) (see Methods for details), using the *respR* package in R (Harianto et al., 2019)
52	Confirm that volume (or mass) of the animal was subtracted from respirometer volume when calculating oxygen uptake rates	Yes
53	Specify whether variation in body mass was accounted for in analyses and describe any allometric body-mass-correction or adjustment	SMR is reported as a mass-specific metabolic rate, whereby body mass did not significantly differ amongst treatment groups (cold *v.* control, p=0.611; warm *v.* control, p=0.876; see Table 1 for details). No allometric scaling exponent was used in the analyses

Table S3: Temperatures in the wrasse (*Ctenolabrus rupestris*) holding tank and water baths before and after immersions.

Date (day-month-year)	Time	Temperature (°C)							
		Tank		Cold bath		Control bath		Warm bath	
		Before	After	Before	After	Before	After	Before	After
15-06-2022	16:30	16.6	-	8.2	-	16.6	-	25.0	-
	19:30	16.7	-	9.6	-	16.7	-	24.5	-
16-06-2022	7:30	16.8	-	8.6	9.2	17.0	17.0	24.7	24.5
	10:30	16.9	-	8.7	9.0	17.0	17.0	24.9	24.5
	13:30	16.6	-	8.7	9.1	16.6	16.2	24.6	24.2
	16:30	16.3	-	8.2	8.5	16.2	16.2	24.2	23.7
	19:30	16.0	-	8.1	8.7	16.0	16.0	24.0	23.5
17-06-2022	7:30	16.1	-	8.1	8.6	16.2	16.2	23.9	23.5
	10:30	16.1	16.1	8.3	8.8	16.0	16.0	24.1	23.7
	13:30	16.2	16.2	8.4	8.9	16.2	16.2	23.9	23.4
	16:30	16.1	16.2	8.1	8.6	16.0	16.0	24.1	23.5
	19:30	16.4	16.4	8.3	8.8	16.3	16.3	24.3	23.8
18-06-2022	7:30	16.1	16.1	8.0	8.5	16.0	16.0	23.9	23.6
	10:30	16.2	16.2	8.1	8.6	16.0	16.1	24.3	23.8
	13:30	16.1	16.3	8.1	8.7	16.1	16.1	24.2	23.8
	16:30	16.3	16.4	8.3	8.8	16.0	16.1	24.3	23.9
	19:30	16.3	16.3	8.3	8.7	16.0	16.1	24.5	24.0
19-06-2022	7:30	16.2	16.3	8.3	8.8	16.2	16.2	24.2	23.8
	10:30	16.2	16.2	8.2	8.8	16.1	16.1	24.2	23.7
	13:30	16.2	16.3	8.2	8.2	16.2	16.1	24.2	23.8
	16:30	16.2	16.2	8.3	8.8	16.1	16.2	24.2	23.7
	19:30	16.3	16.3	8.2	8.7	16.3	16.2	24.3	23.9
20-06-2022	7:30	16.2	16.2	8.2	8.8	16.3	16.3	24.2	23.8
	10:30	16.3	16.3	8.2	8.6	16.3	16.3	24.3	23.8
	13:30	16.3	16.3	8.2	8.7	16.3	16.3	24.4	24.0
	16:30	16.3	16.4	8.4	8.9	16.1	16.1	24.3	23.8
